# Geographical Distribution of β-Lactam Resistance among *Klebsiella spp.* from Selected Health Facilities in Ghana

**DOI:** 10.3390/tropicalmed4030117

**Published:** 2019-09-03

**Authors:** Elvis Quansah, Prince Amoah Barnie, Desmond Omane Acheampong, Dorcas Obiri-Yeboah, Richael Odarkor Mills, Ebenezer Asmah, Obed Cudjoe, Isaac Dadzie

**Affiliations:** 1 Department of Microbiology and Immunology, School of Medical Sciences, University of Cape Coast, Cape Coast, Ghana (E.Q.) (D.O.-Y.) (E.A.) (O.C.); 2 Department of Forensic Sciences, School of Biological Sciences, University of Cape Coast, Cape Coast, Ghana; 3 Department of Biomedical Sciences, School of Allied Health Sciences, University of Cape Coast, Cape Coast, Ghana (D.O.A.) (R.O.M.); 4 Department of Medical Laboratory Sciences, School of Allied Health Sciences, University of Cape Coast, Cape Coast, Ghana

**Keywords:** β-lactamases, *Klebsiella spp.*, extended spectrum beta-lactamase, AmpC, carbapenemase, metallo-β-lactamase

## Abstract

β-Lactam-resistant *Klebsiella* isolates continue to cause multidrug resistance infections worldwide. This study aimed to describe the geographical distribution of extended spectrum β-lactamase (ESBL), AmpC β-lactamase (AmpC), and carbapenemase production among 139 *Klebsiella* isolates recovered from patients at major referral health facilities in Ghana. The phenotypic methods of combined disc diffusion test, modified three-dimensional test, modified Hodge test (MHT), and combined disc test were performed for each isolate to detect ESBL, AmpC, carbapenemase, and metallo-β-lactamase (MBL) producers, respectively. Except for MBL, all other β-lactam resistance mechanisms were highest in the healthcare facilities situated in the northern belt of Ghana. Significant regional difference of ESBL producers was observed between the northern and middle belts as well as the northern and southern belts. Genotypic detection with polymerase chain reaction (PCR) revealed the presence of *bla* TEM 36/139 (25.9%), *bla* SHV 40/139 (28.8%), *bla* CTX-M 37/139 (26.6%), *bla* OXA-48 3/139 (2.16%), and *bla* NDM 1/139 (0.72%) genotypes. In conclusion, there were variations in β-lactam resistance among *Klebsiella spp*. from health facilities situated in the northern, middle, and southern belts of Ghana. The study provides preliminary evidence that emphasizes the need to direct more attention to antimicrobial resistance control, especially in the northern belt of Ghana. Findings from this study may be critical for creating and fine-tuning effective antimicrobial resistance control strategies and for informing accurate antibiotic prescription by practitioners.

## 1. Introduction

Among the human infections caused by the genus *Klebsiella, Klebsiella pneumoniae* remain the most clinically relevant species, accounting for over 70% of these infections [1]. Also, recent evidence indicates increasing relevance of *Klebsiella oxytoca* in nosocomial infections [2]. In major referral health facilities in Ghana, *K. pneumoniae* is among the top three most frequently isolated Gram-negative bacteria that are primarily recovered from patients with urinary tract infection, respiratory tract infection, wound infections, and sepsis [3,4,5]. 

The emergence of β-lactamases, including extended spectrum β-lactamases (ESBLs), AmpC-β-lactamases (AmpCs), and carbapenemases, particularly in *Klebsiella spp.*, is a central health concern [6]. Until the 1990s, *K. pneumoniae* typically produced a single or rarely two plasmid-encoded β-lactamases. Today, *K. pneumoniae* harboring more than five β-lactamases are commonplace in some clinical facilities [7]. *K. pneumonia* is now recognized as “a pool of potent β-lactamases” [8] and an indicator species for plasmid-encoding β-lactamases [8]. These β-lactamases constitute the chief resistance mechanisms that mediate the inactivation of β-lactam antibiotics by hydrolyzing the amide bond of the β-lactam ring [9,10]. Of all carbapenemases, metallo-β-lactamase (MBL) are the most transmissible and are particularly troublesome owing to their recent global dissemination and enhanced ability to hydrolyze a wide range of β-lactams [11]. 

Recent findings in neighboring West African countries have indicated worrying levels of ESBLs in *Klebsiella* isolates relative to other Enterobacteriaceae [12,13]. More worrying is the recent increasing documentation of high levels of carbapenemase-encoding genes in *K. pneumoniae* in a number of African countries [14,15,16]. Although a modest number of studies have documented high levels of ESBL production and AmpC production in *Klebsiella* isolates in Ghana [17,18,19,20], studies directed at the exploration of carbapenemase production among *Klebsiella spp*. is very limited. 

The northern, middle, and southern belts of Ghana markedly differ in terms of temperature and socioeconomic status [21,22]. Temperature and socioeconomic status are posited to be key determinants of antimicrobial resistance [23,24]. Nonetheless, precise data on the regional distribution of these β-lactam resistance mechanisms among *Klebsiella* isolates in Ghana seems lacking, preventing the identification of key areas that may need very robust antimicrobial resistance control strategies. Against this background, this study aimed to describe the geographical distribution of ESBL-, AmpC-, and carbapenemase-producing *Klebsiella* isolates from major referral health centers situated in the northern, middle, and southern belts of Ghana using phenotypic and genotypic techniques. The results from this study will provide a preliminary local evidence of regions in Ghana that may need robust antimicrobial resistance control strategies. 

## 2. Materials and Method

### 2.1. Collection and Identification of Isolates

Nonrepetitive isolates were collected from October 2017 to March 2018. To ensure fair representation, healthcare facilities that serve as referral centers in the northern, middle, and southern belts of Ghana were selected for the study. *Klebsiella* isolates were obtained from Tamale Teaching Hospital and Bolgatanga Regional Hospital (northern belt), Komfo Anokye Teaching Hospital (middle belt) and Cape Coast Teaching Hospital and Effia Nkwanta Regional Hospital (southern belt). These isolates were transported in Luria–Bertani (LB) broth (Oxoid, Basingstoke Hampshire, UK) to the clinical microbiology laboratory (Department of Biomedical Science, University of Cape Coast, Cape Coast, Ghana) for investigation. Isolates were subcultured on McConkey agar media (Oxoid, Basingstoke Hampshire, UK). All the isolates were reidentified using Gram staining and a panel of biochemical tests [25]. 

### 2.2. Phenotypic Techniques

#### Antimicrobial Susceptibility Testing (AST)

Antimicrobial susceptibility testing (AST) was carried out on Mueller–Hinton agar (Techno Pharm Chem, Delhi, India) and interpreted according to the Clinical and Laboratory Standards Institute (CLSI) guidelines [26]. The antimicrobial susceptibility testing was performed using ampicillin (10 µg), tetracycline (10 µg), cotrimoxazole (25 µg), gentamicin (10 µg), cefuroxime (30 µg), chloramphenicol (10 µg), ceftriaxone (30 µg), cefotaxime (30 µg), ciprofloxacin (5 µg), amikacin (30 µg), meropenem (10 µg), imipenem (10 µg), ertapenem (10 µg), cefoxitin (30 µg), and cefotetan (30 µg) (BIO-RAD, Munich, Germany). *Escherichia Coli* ATCC 25299 was used as a quality control strain. 

Detection of ESBL production was carried out on a Mueller–Hinton agar (Oxoid, Basingstoke Hampshire, UK) plate seeded with the test isolate. Combined discs of either cefotaxime (30 µg)/clavulanic acid (10 µg) or ceftazidime (30 µg)/clavulanic acid (10 µg) (BIO-RAD, Munich, Germany) was placed 20 mm away from single discs of cefotaxime (30 µg) or ceftazidime (30 µg), respectively, and incubated at 37 °C overnight. An enhanced zone of inhibition (>5 mm) around the combined disc relative to the single disc was considered positive for the production of ESBL [27]. *Escherichia Coli* ATCC 25299 was used as a negative control strain, whereas *K. pneumoniae* ATCC 700603 were used as positive control. 

Isolates that were resistant to either cefoxitin (30 µg), cefotetan (30 µg), or both after antimicrobial susceptibility screening were selected for confirmation of AmpC production using modified three-dimension test. The procedure described by Deotale et al. [28] was used. The positive test was read as a small heart-shaped indentation toward the cefoxitin or cefotetan disc seen at the junction of the slit along the line of inhibition. 

Isolates resistant to the carbapenem-based drugs were screened for carbapenemase production using modified Hodge test, as recommended by CLSI [26]. A total of four organisms were tested on each plate. A clover leaf-type indentation at the intersection of the test organism and *Escherichia Coli* ATCC 25299 within the zone of inhibition around the carbapenem disc was considered positive for carbapenemase production. *K. pneumoniae* ATCC BAA-1705 and *Escherichia Coli* ATCC 25299 was used as the positive and negative control, respectively. 

The isolates that were resistant to meropenem, ertapenem, or imipenem were further tested for MBL production using the combined disc method. Carbapenem/ethylenediaminetetraacetic acid (EDTA) (10/930 µg) combined discs (BIORAD, Munich, Germany) of meropenem + EDTA, ertapenem + EDTA, and imipenem + EDTA were placed 20 mm from 10 µg discs of meropenem, ertapenem, and imipenem, respectively. An increased inhibition zone size ≥7 mm with carbapenem/EDTA was compared to the carbapenem discs alone and was taken as MBL-positive [29]. 

### 2.3. Genotypic Techniques

Bacterial DNA extraction was carried out using the crude boiling method. Bacterial isolates were subcultured on LB agar (Oxoid, Basingstoke Hampshire, UK) and incubated overnight. Isolate (1–2 colonies) were picked and emulsified in 100 µL of sterile distilled water contained in a 1.5 mL sterile Eppendorf tube. Samples were then boiled for 10 min at 100 °C to allow for cell lysis. The lysates were then centrifuged at 4000 rpm for 5 min at 4 °C. The supernatant was transferred into a new tube, and 3 µL of this served as the template for subsequent polymerase chain reaction (PCR) reactions. 

Selected ESBL (TEM, SHV, CTX-M) and carbapenemase-encoding genes (OXA-48, NDM, IMP, VIM, and KPC) were detected by PCR for all isolates with primers (Sangong, Shanghai, China) listed in Table 1. PCR was carried out using thermal cycler (BioRad, Hercules, CA, USA) with a total reaction volume of 20.0 µL containing 3.0 µL DNA template and 4.0 µL of Tag 5X Master Mix (New England Biolabs), 0.5 µL MgCl_2_, 0.7 µL of 10 μmol L^−1^ forward primer, 0.7 µL of μmol L^−1^ reverse primer, and 11.1 µL ddH_2_O. Cyclin condition for SHV, CTX-M, KPC, NDM, IMP, VIM, and OXA48 were set at 95 °C for initial denaturation for 5 min. The second step included 35 cycles of denaturation at 95 °C for 30 s, annealing at 55 °C for 45 s, and elongation at 72 °C for 45 s. This was followed by a final extension temperature of 72 °C for 7 min. Cyclin conditions for TEM was set at 95 °C for initial denaturation for 5 min. The second step included 35 cycles of denaturation at 95 °C for 1 min, annealing at 55 °C for 1 min, and elongation at 72 °C for 1 min. ESBL and carbapenemase controls described elsewhere were used [14,30]. Amplified products were detected by electrophoresis using 1% agarose gel in Tris-acetate-EDTA (TAE) buffer and visualized with ethidium bromide staining (BDH Prolabo, Leuven, Belgium). 

### 2.4. Data Analysis

Chi-square analysis was used to compare categorical variables. Fisher’s exact test was used for cells with expected count below 5. All analyses were done using IBM statistical package for social sciences (SPSS) version 21 (IBM corporation, New York, NY, USA).

## 3. Results

### 3.1. Study Population

The study identified 139 *Klebsiella* isolates, of which 70/139 (50.4%), 21/139 (15.1%), 26/139 (18.7%), 10/139 (7.1%), and 12/139 (8.6%) isolates were obtained from Komfo Anokye Teaching Hospital, Cape Coast Teaching Hospital, Effia Nkwanta Regional Hospital, Tamale Teaching Hospital, and Bolgatanga Regional Hospital, respectively. Of the 139 isolates, 48 (34.5%) were *K. oxytoca*, whereas 91 (65.5%) were *K. pneumoniae.* The isolates were mainly recovered from blood 18/139 (12.9%), high vaginal swab (HVS) 12/139 (8.6%), sputum 20/139 (14.4%), urine 63/139 (45.3), wound 18/139 (12.9), and “others” [8/139 (5.8%) ear swabs, pleural aspirates, urethral swab, tracheal aspirates, and cholestetial] (Table 2). 

### 3.2. Antimicrobial Susceptibility Profile of Isolates

The isolates were tested against a panel of 16 antibiotics comprising 10 β-lactam- and six non-β-lactam-based drugs. High resistance to ampicillin was observed for *K. oxytoca* 47/48 (97.9%) and *K. pneumoniae* 91/91 (100%). There was also a greater than 70% resistance against the 3rd-generation cephalosporins (ceftriaxone, cefotaxime, and ceftazidime) by both species. The carbapenem-based drugs were relatively active compared to the other classes of β-lactam drugs. Among the carbapenem-based drugs, meropenem was the least active agent, recording 18/48 (37.5%) and 27/91 (29.7%) resistance against *K. oxytoca* and *K. pneumoniae*, respectively. The most active carbapenem agent was imipenem, producing 5/48 (10.4%) and 13/91 (14.3%) resistance against *K. oxytoca* and *K. pneumoniae*, respectively. The most active non-β-lactam was amikacin. Except for amikacin, to which just 1/48 (2.1%) *K. oxytoca* and 4/91 (4.4%) *K. pneumoniae* showed resistance, high resistance to the other non-β-lactam antibiotics was recorded for *K. oxytoca* (54.2%–89.6%) and *K. pneumoniae* (73.6%–91.2%) (Table 3). 

### 3.3. Phenotypic Distribution of ESBL-, AmpC-, MBL-, and Carbapenemase-Producing Klebsiella Isolate by Specimen Type

There was no evidence of any association (*p* > 0.005) between ESBL, AmpC, and MBL production and the species of *Klebsiella* (Table 4). On the other hand, the proportion of carbapenemase producers within *K. oxytoca* species 6/48 (12.5%) was significantly (*p* < 0.05) lower compared to the proportion of carbapenemase producers within *K. pneumoniae* species 24/91 (26.4%). ESBL producers were most abundant in “others” [6/8 (75.0%), ear swabs, pleural aspirates, urethral swab, tracheal aspirates, and cholestetial fluid] and least abundant in HVS 7/12 (58.3%). HVS 5/12 (41.7%) represented the specimen type that harbored the most AmpC producers, whereas sputum, wound swab, and “others” recorded no (0%) AmpC producers. MBL producers were predominantly recovered from HVS 4/12 (33.3%). However, wound swab 5/18 (27.8%) served as the source that harboured the most carbapenemase producers. Details of the distribution of β-lactamase producers of *K. oxytoca* and *K. pneumoniae* are presented in Table 4.

### 3.4. Phenotypic Distribution of ESBL-, AmpC-, and Carbapenemase-Producing Klebsiella Species by Geographical Location 

A geographical decreasing gradient of ESBL and carbapenemase production was observed from the northern belt to the southern belt as opposed to an increasing gradient of MBL production from the northern to the southern belt. The production of AmpC was highest in the northern belt, followed by the southern and middle belts. 

**Figure 1:**
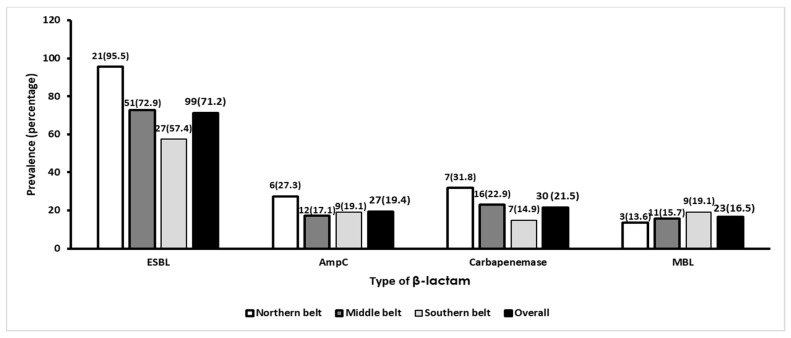
Phenotypic distribution of ESBL-, AmpC-, carbapenemase-, and MBL-producing *Klebsiella* isolates from referral health facilities in Ghana.

The overall carbapenemase producers were found to be 30/139 (21.5%). The highest resistance mechanism among the *Klebsiella* isolate was ESBL 99/139 (71.2%), followed by MBL 23/139 (16.5%), and AmpC 27/139 (19.4%). The ESBL resistance observed at health facilities in the northern belt significantly differed from that of the middle belt (*p* = 0.012) and southern belt (*p* = 0.001) but not between the middle and southern belts. There were no significant regional differences in the production of AmpC, carbapenemase, and MBL (*p* > 0.05). Figure 1 gives a summary of the geographical distribution of ESBL, AmpC, carbapenemase, and MBL production among *Klebsiella* isolates from health facilities in Ghana. 

### 3.5. Genotypic Distribution of bla ESBL-Encoding Genes in Klebsiella Species 

There was no evidence of association (*p* > 0.05) between the occurrence of *bla* ESBL genes (TEM, SHV, CTX-M) and the *Klebsiella* species. However, the proportion of TEM-positive *Klebsiella* isolates from the facilities within the northern belt 9/22 (40.9) was significantly higher (*p* = 0.032) than the proportion of TEM-positive *Klebsiella* isolate from health facilities within the southern belt 8/47 (17.0%). Furthermore, the proportion of SHV-positive isolates in the health facility at the middle belt 25/70 (35.7%) was significantly higher (*p* = 0.053) than the proportion of SHV-positive isolates from health facilities within the southern belt 9/47 (19.1%). *bla* TEM, *bla* SHV, and *bla* CTX-M were most abundant in sputum 7/20 (35.0%), HVS 4/12 (33.3%) and blood 6/18 (33.3%), respectively. Details of the distribution of *bla* ESBL genes by *Klebsiella* species, geographical location of health facility, and specimen are presented in Table 5. 

### 3.6. Distribution of Blacarbapenemase-Positive Isolates

A total of 3/139 (2.1%) isolates were identified as positive for carbapenemase genes. Of the three isolates, one was positive for harboring two carbapenemase genes, i.e., OXA-48- and NDM-encoding genes, whereas the other two were positive for only OXA-48-encoding genes. No isolate was positive for IMP, VIM, and KPC. Two of the positive isolates were recovered from facilities situated in the southern belt, whereas the remaining one was isolated in the middle belt. Table 6 shows the details of isolates positive for carbapenemase-encoding genes and their respective antibiogram. 

### 3.7. Agreement between Phenotypic Tests and Genotypic Test 

Of the 72 genotypically positive ESBL isolates, only 51 (70.8%) were phenotypically positive. Of the 21 apparent false-negative ESBLs, 12 were phenotypically positive for AmpC production. All three genotypically carbapenemase-positive isolates were negative for MHT. However, two (EF 41 and EF I41) were positive for MBL production.

## 4. Discussion

*Klebsiella spp*. are an important cause of multidrug-resistant infections with varying reports of β-lactam antimicrobial resistance levels worldwide. This study described the distribution of ESBL, AmpC, carbapenemase, and MBL resistance mechanisms by geographical location of the health facilities in Ghana. 

The most recent nationwide antimicrobial resistance survey in Ghana involving a broad spectrum of bacteria isolates showed that isolates from healthcare facilities in the northern belt had generally higher resistance profile compared to the middle and southern belts [37]. The investigators of the survey found a statistically significant (*p* < 0.05) difference in antimicrobial resistance levels between isolates from the northern and southern belts. Likewise, the present study documented the highest prevalence of ESBL, AmpC, and carbapenemase production in *Klebsiella* isolates from health facilities situated within the northern belt. Similar to the nationwide survey, the present study reported a significantly (*p* < 0.05) higher ESBL production in the northern belt compared to not only the southern belt but also the middle belt. This may be a reflection of the relatively higher genotypic levels of TEM and CTX-M in the northern belt, as shown by this study. 

The prevalence of ESBL, carbapenemase, and MBL producers recorded in the healthcare facility in the middle belt was in between that recorded in the northern and southern belts. This could reflect the arbitrary moderate temperatures and socioeconomic burden in the middle belt of Ghana. Notably, isolates positive for carbapenemase-encoding genes were detected in facilities situated in the middle belt (one isolate) and southern belt (two isolates) but none in the northern belt. This contradicts the phenotypic finding that indicated a higher prevalence of carbapenemase production in the northern belt. Perhaps the presence of other resistance mechanism or carbapenemase-resistant genes not explored by this study could explain these two paradoxical findings. The factors underlining the observed varying levels of β-lactam resistance mechanisms could be accounted for by the difference in temperatures and socioeconomic status in these regions. It is postulated that high levels of resistant pathogens correlate with regions with warmer temperatures [23,24]. In the case of *K. pneumoniae*, it is estimated that a 10 °C change in temperature is associated with a 2.2% increase in resistance [23]. One model aimed at assessing the vulnerability of Ghana with respect to climate change estimated a temperature increase of 1.5 to 3.0 °C by 2060, with the northern belt expected to be the most affected zone [38]. In Ghana, the northern belt is the warmest belt [22], and this could contribute to the high resistance observed in the north. In addition, antimicrobial resistance-driving factors, including self-medication, prescription of substandard antimicrobials, and poor sanitation and hygiene have been shown to be associated with low socioeconomic status [39,40]. The northern belt of Ghana is deemed the poorest of the three belts in terms of economic status [21], and this could partly contribute to the relatively high resistance observed in this region. 

A recent study documented the presence of carbapenem resistance in 2.9% of Gram-negative bacteria [41]. However, in the present study, not all the carbapenem-resistant isolates tested positive with MHT, suggesting that more than 30 isolates (>21.5%) are resistant to one or more carbapenem drugs. The reports from these two studies are worrisome because, in Ghana, carbapenems are usually reserved for the treatment of serious infections, and as such resistance to carbapenems is expected to be low. The difference between these two reports could be explained by the fact that the prevalence estimated by Codjoe et al. was among a broad-spectrum Gram-negative isolates, whereas the one estimated by the present study was the prevalence among *Klebsiella* isolates alone. Also, the difference in sample size might have partly contributed to the discrepancies between these two studies. Codjoe and his colleagues identified two OXA-48-encoding genes in *K. pneumoniae* and NDM-encoding genes in *Pseudomonas aeruginosa* and *Acinetobacter spp.* [41]. In concordance with this earlier study [41], three OXA-48 carbapenemase-positive *Klebsiella* isolates were identified by the present study, of which one was also positive for NDM-encoding gene. Findings from this study, together with the findings of Codjoe et al. [41], might suggest a possible ongoing interspecies dissemination of NDM in Ghana. *K. pneumoniae* coharboring NDM and OXA-48 have also been previously documented in Tunisia and Uganda [14,42]. This indicates how common *K. pneumoniae* isolates coharboring NDM and OXA-48 are reported in Africa. 

The study recorded 21/72 false-negative ESBL production, of which 12 were phenotypically positive for AmpC production. This could be due to the concomitant expression of ESBL and plasmid-mediated AmpC [43]. In addition, of the 99 phenotypically positive ESBL isolates, only 51 were genotypically positive for ESBL-encoding genes. This suggests a possible resistance by non-ESBL genes or the presence of other possible atypical ESBL genes, which were not explored by the study. All three genotypically positive carbapenemase producers were negative with MHT, reiterating the poor performance of MHT. However, isolate EF-41 harboring NDM and OXA-48 was identified as positive with MBL test. In addition, EF-141 (positive for only OXA-48 carbapenemase) was also positive for MBL test, suggesting the presence of other possible MBL genes not explored by the present study. 

The findings of this study remain observational as factors including the limited number of isolates used, the restricted choice of bacteria isolates, and the short period within which the study was carried out might have potentially affected the outcome. For instance, the sample size in the present study, especially in the northern belt, might not be adequately powered to estimate the geographical differences in certain β-lactamases, including carbapenemase and AmpC. Another limitation is that the presence of specific AmpC-resistant genes was not tested by the present study. 

## 5. Conclusions

There were variations in β-lactam resistance among *Klebsiella spp*. from health facilities situated in the northern, middle, and southern belts of Ghana. β-Lactam resistance at the health facilities situated within the northern belt was considerably higher compared to health facilities located within the middle and southern belts. The detection of OXA-48- and NDM-resistant genes could signal the commencement of carbapenemase dissemination, adding to the already alarmingly high prevalence of ESBL-specific genes in Ghana. This study provides preliminary evidence that emphasizes the need to direct more attention to antimicrobial resistance monitoring and surveillance, especially in the northern belt of Ghana. This would be critical for creating and fine-tuning effective antimicrobial resistance control strategies and for informing accurate antibiotic prescription in clinical settings.

## 6. Future Perspective

This study serves as a baseline study for further studies targeting different bacteria isolates and different antimicrobial resistance mechanisms. Although the study provides insight into the phenotypic and genotypic prevalence of ESBL, AmpC, and carbapenemase and their regional distribution in *Klebsiella* isolates, little work has been done to molecularly characterize carbapenemase genes in Ghana. Future perspective may include molecular characterization of NDM- and OXA-48-producing *K. pneumoniae* in Ghana. In addition, molecular testing could be performed to characterize the resistance profile to amikacin against *Klebsiella* isolates. 

## Figures and Tables

**Table 1:** Primer sequence used for amplification of extended spectrum β-lactamase (ESBL) and carbapenemase genes.

Name	Primer Sequence (5’→3’)	Amplicon Size (bp)	Reference
TEM	Forward-ATGAGTATTCAACATTTCCG Reverse-TTACCAATGCTTAATCAGTGAG	861	[31]
SHV	Forward-TCAGCGAAAAACACCTTG Reverse-TCCCGCAGATAAATCACCA	472	[31]
CTX-M	Forward-GCGATGGGCAGTACCAGTAA Reverse-TTACCCAGCGTCAGATTCCG	392	[31]
KPC	Forward-CATTCAAGGGCTTTCTTGCTGC Reverse-ACGACGGCATAGTCATTTGC	538	[32]
NDM	Forward-GGTTTGGCGATCTGGTTTTC Reverse-CGGAATGGCTCATCACGATC	521	[33]
IMP	Forward-TTGACACTCCATTTACAG Reverse-GATTGAGAATTAAGCCACTCT	232	[34]
VIM	Forward-TTATGGAGCAACCGATGT Reverse-CAAAAGTCCCGCTCCAACGA	920	[35]
OXA-48	Forward-GCTTGATCGCCCTCGATT Reverse-GATTTGCTCCGTTGGCCAAA	281	[36]

**Table 2:** Frequency of *Klebsiella* isolates from different specimen types at the five different hospitals across Ghana.

Facility	Specimen n (%)						Total
	Blood	HVS	Sputum	Urine	Wound Swab	Others *	
Northern belt							
TTH							
*Klebsiella oxytoca*	0	0	2 (66.7)	2 (50.0)	0	0	4 (40.0)
*Klebsiella pneumoniae*	1 (100)	1 (100)	1 (33.3)	2 (50.0)	0	1 (100)	6 (60.0)
BRH							
*Klebsiella oxytoca*	0	0	0	3 (30.0)	0	1 (50.0)	4 (33.3)
*Klebsiella pneumoniae*	0	0	0	7 (70.0)	0	1 (50.0)	8 (66.7)
Middle belt							
KATH							
*Klebsiella oxytoca*	3 (21.4)	0	2 (20.0)	13 (39.4)	2 (20.0)	0	20 (28.6)
*Klebsiella pneumoniae*	11 (78.6)	0	8 (80.0)	20 (60.6)	8 (80.0)	3 (100)	50 (71.4)
Southern belt							
CCTH							
*Klebsiella oxytoca*	1 (33.3)	3 (100)	0	3 (37.5)	3 (50.0)	0	10 (47.6)
*Klebsiella pneumoniae*	2 (66.7)	0	0	5 (62.5)	3 (50.0)	1 (100)	11 (52.4)
EFNTH							
*Klebsiella oxytoca*	0	2 (25.0)	3 (42.9)	3 (37.5)	2 (100)	0	10 (38.5)
*Klebsiella pneumoniae*	0	6 (75.0)	4 (57.1)	5 (62.5)	0	1 (100)	16 (61.5)
Total	18 (100)	12 (100)	20 (100)	63 (100)	18 (100)	8 (100)	139 (100)

KATH = Komfo Anokye Teaching Hospital, CCTH = Cape Coast Teaching Hospital, EFNTH = Effia Nkwanta Teaching Hospital, TTH = Tamale Teaching Hospital, BRH = Bolgatanga Regional Hospital. Others* = ear swabs, pleural aspirates, urethral swab, tracheal aspirates, and cholestetial fluid.

**Table 3:** Antimicrobial susceptibility profile of *Klebsiella oxytoca* and *Klebsiella pneumoniae*.

Variable	*K. oxytoca* (*n* = 48)			*p*-Value	*K. pneumoniae* (*n* = 91)			*p*-Value
	R n (%)	I n (%)	S n (%)		R n (%)	I n (%)	S n (%)	
β-lactams								
Penicillin								
Ampicillin	47 (97.9)	1 (2.1)	0 (0.0)	<0.001	91 (100)	0 (0.0)	0 (0.0)	<0.001
3rd-gen cephalosporin								
Ceftriaxone	40 (83.3)	3 (6.3)	5 (10.4)	<0.001	83 (91.2)	7 (7.7)	1 (1.1)	<0.001
Cefotaxime	44 (91.7)	2 (4.2)	2 (4.2)	<0.001	70 (76.9)	8 (8.8)	13 (14.3)	<0.001
Ceftazidime	38 (79.2)	4 (8.3)	6 (12.5)	<0.001	65 (71.4)	15 (16.5)	11 (12.1)	<0.001
2nd-gen cephalosporin								
Cefuroxime	42 (87.5)	3 (6.3)	3 (6.3)	<0.001	90 (98.9)	1 (2.1)	0 (0.0)	<0.001
Cefoxitin	18 (37.5)	11 (22.9)	19 (39.5)	0.168	31 (36.5)	24 (28.2)	30 (35.3)	0.480
Cefotetan	14 (29.2)	1 (2.1)	33 (68.8)	<0.001	18 (19.8)	4 (4.4)	69 (75.8)	<0.001
Carbapenem								
Meropenem	18 (37.5)	9 (18.8)	21 (43.8)	0.026	27 (29.7)	25 (27.5)	39 (42.9)	0.059
Imipenem	5 (10.4)	0(0.0)	43 (89.6)	<0.001	13 (14.3)	1 (1.1)	77 (84.6)	<0.001
Ertapenem	7 (14.6)	9 (18.8)	32 (66.7)	<0.001	15 (16.5)	14 (15.4)	62 (68.1)	<0.001
Non-β-lactams								
Tetracycline	41 (85.4)	3 (6.3)	4 (8.3)	<0.001	77 (84.6)	1 (1.1)	13 (14.3)	<0.001
Cotrimoxazole	43 (89.6)	-	5 (10.4)	<0.001	83 (91.2)	0 (0.0)	8 (8.8)	<0.001
Ciprofloxacin	33 (68.8)	15 (31.3)	0 (0.0)	<0.001	69 (75.8)	22 (24.2)	0 (0.0)	<0.001
Chloramphenicol	26 (54.2)	4 (8.3)	18 (37.5)	<0.001	67 (73.6)	4 (4.4)	20 (22)	<0.001
Gentamicin	31 (64.6)	4 (8.3)	13 (27.1)	<0.001	67 (73.6)	3 (3.3)	21 (23.1)	<0.001
Amikacin	1 (2.1)	0 (0.0)	47 (97.9)	<0.001	4 (4.4)	0 (0.0)	87 (95.6)	<0.001

**Table 4:** Prevalence of ESBL-, AmpC β-lactamase (AmpC)-, metallo-β-lactamase (MBL)- and carbapenemase-producing *Klebsiella species* by specimen, Ghana.

Resistance Mechanism	Species	Within Species	Total	Blood *n* = 18	HVS *n* = 12	Sputum *n* = 20	Urine *n* = 63	Wound Swab *n* = 18	Others *n* = 8
ESBL producers *n* = 99	K.O	35/48 (72.9) ^#^	35/99 (35.4)	4/4 (100)	2/5 (40.0)	5/7 (71.4)	17/24 (70.8)	6/7 (85.7)	1/1 (100)
	K.P	64/91 (70.3)	64/99 (64.6)	9/14 (64.2)	5/7 (71.4)	9/13 (69.2)	29/39 (74.3)	7/11 (63.6)	5/7 (71.4)
Overall prevalence (%)		99/139 (71.2)	(100)	13/18 (72.2)	7/12 (58.3)	14/20 (70.0)	46/63 (73.0)	13/18 (72.2)	6/8 (75.0)
AmpC producers *n* = 27	K.O	8/48 (16.6) ^#^	8/27 (29.6)	3/4 (75.0)	2/5 (40.0)	0/7 (0)	3/24 (12.5)	0/7 (0)	0/1 (0)
	K.P	19/91 (20.8)	19/27 (70.4)	1/14 (7.1)	3/7 (42.9)	5/13 (38.5)	7/39 (17.9)	2/11 (18.1)	1/7 (14.3)
Overall prevalence (%)		27/139 (19.4)	(100)	4/18 (22.2)	5/12 (41.7)	5/20 (25.0)	10/63 (15.9)	2/18 (11.1)	1/8 (12.5)
MBL producers *n* = 23	K.O	5/48 (10.4) ^#^	5/23 (21.7)	0/4	1/5 (20.0)	2/7 (28.6)	1/24 (4.1)	1/7 (14.3)	0/1
	K.P	18/91 (19.8)	18/23 (78.3)	3/14 (21.1)	3/7 (42.9)	3/13 (23.1)	3/39 (7.7)	4/11 (36.4)	2/7 (28.6)
Overall prevalence (%)		23/139 (16.5)	(100)	3/18 (16.7)	4/12 (33.3)	5/20 (25.0)	4/63 (6.3)	5/18 (27.8)	2/8 (25.0)
Carbapenemase producers *n* = 30	K.O	6/48 (12.5) *	6/30 (20.0)	0/4 (0)	1/5 (20.0)	1/7 (14.3)	4/24 (16.7)	0/7 (0)	0/1 (0)
	K.P	24/91 (26.4)	24/30 (80.0)	4/14 (28.6)	3/7(42.9)	4/13 (30.8)	12/39 (30.8)	5/11 (45.5)	2/7 (28.6)
Overall prevalence (%)		30/139 (21.6)	(100)	4/18 (22.2)	4/12 (33.3)	5/20 (25.0)	16 (25.4)	5/18 (27.8)	2/8 (25.0)

K.O = *Klebsiella oxytoca,* K.P = *Klebsiella pneumoniae*, Total = producers in a particular species expressed as a percentage (%) of the total number of producers in the study, HVS = high vagina swab. Chi-square analysis was used to compare all categorical variables at an alpha value of 0.05. Fisher’s exact test was used for cells with counts less than 5. Data is presented as count (%) ^#^ = no significant difference comparing producers of K.O and K.P., * = significant difference comparing producers of K.O and K.P. Others = ear swabs, pleural aspirates, urethral swab, tracheal aspirates, and cholestetial fluid.

**Table 5:** Distribution of *bla* ESBL genes by *Klebsiella* species and geographical location.

Variable	*Bla*ESBL		
	TEM (*n* = 36)	SHV (*n* = 40)	CTX-M (*n* = 37)
Species			
*Klebsiella oxytoca* (*n* = 48)	11/48 (22.9%)	13/48 (27.1%)	10/48 (20.8%)
*Klebsiella pneumoniae* (*n* = 91)	25/91 (27.5%)	27/91 (29.7%)	27/91 (29.7%)
*p*-Value^a^	0.560	0.749	0.262
Region			
Northern belt (*n* = 22)	9/22 (40.9%)	6/22 (27.3%)	7/22 (31.8%)
Middle belt (*n* = 70)	19/70 (27.1%)	25/70 (35.7%)	22/70 (31.4%)
Southern belt (*n* = 47)	8/47 (17.0%)	9/47 (19.1%)	8/47 (17.0%)
*p*-Value^b^	0.221	0.465	0.973
*p*-Value^c^	0.032	0.446	0.165
*p*-Value^d^	0.203	0.053	0.080
Specimen			
Blood (*n* = 18)	3/18 (16.7%)	5/18 (27.8%)	6/18 (33.3%)
HVS (*n* = 12)	2/12 (16.7%)	4/12 (33.3%)	3/12 (25.0%)
Others (*n* = 8)	1/8 (12.5%)	2/8 (25.0%)	2/8 (25.0%)
Sputum (*n* = 20)	7/20 (35.0%)	6/20 (30.0%)	3/20 (15.0%)
Urine (*n* = 63)	18/63 (28.5%)	18/63 (28.5%)	19/63 (30.1%)
Wound swab (*n* = 18)	5/18 (27.7%)	5/18 (27.7%)	4/18 (22.2%)
Total	36/139 (25.9%)	40/139 (28.8%)	37 (26.6%)

Chi-square analysis was used to compare all categorical variables at an alpha value of 0.05. Fisher’s exact test was used for cells with counts less than 5. *p*-Value^a^ = *p*-value for comparing *Klebsiella oxytoca* with *Klebsiella pneumoniae, p*-value^b^ = *p*-value for comparing northern belt with middle belt, *p*-value^c^ = *p*-value for comparing northern belt with southern belt, *p*-value^d^ = *p*-value for comparing middle belt with southern belt.

**Table 6:** Antibiogram of *bla* carbapenemase-positive *Klebsiella* isolates.

Variable	Resistance Profile		
Carbapenemase	OXA-48, NDM	OXA-48	OXA-48
ID	EF 41	EF 141	KATH 05
Presence of ESBL	TEM, CTX-M	SHV	-
Species	*K. pneumoniae*	*K. pneumoniae*	*K. pneumoniae*
Age(years)	73	40	57
Specimen	Urine	Sputum	Sputum
Hospital	Effia Nkwanta Regional Hospital	Effia Nkwanta Regional Hospital	Komfo Anokye Teaching Hospital
Geographical location	Southern belt	Southern belt	Middle belt
β-Lactams			
3rd-gen cephalosporin			
Ceftriaxone	R	R	S
Cefotaxime	R	R	S
Ceftazidime	R	R	R
2nd-gen cephalosporin			
Cefuroxime	R	R	R
Cefoxitin	S	I	S
Cefotetan	S	S	R
*Carbapenems*			
Meropenem	I	R	R
Imipenem	R	R	R
Ertapenem	S	S	R
Non-β-lactams			
Ampicillin	R	R	R
Tetracycline	S	R	R
Cotrimoxazole	R	R	S
Ciprofloxacin	R	R	R
Chloramphenicol	R	R	S
Gentamicin	R	R	R
Amikacin	S	S	S
